# Osteogenic Differentiation in Chitosan-Based Scaffolds via P28 and VEGF Delivery

**DOI:** 10.3390/molecules30173645

**Published:** 2025-09-07

**Authors:** Keran Zhou, Bianca Simonassi-Paiva, Robert Pogue, Emma Murphy, Zhi Cao, Margaret Brennan Fournet, Declan M. Devine

**Affiliations:** 1 PRISM Research Institute, Technological University of the Shannon, Midlands Midwest, Athlone Main Campus, N37 HD68 Athlone, Ireland; a00258808@student.tus.ie (K.Z.);; 2 Genomic Sciences and Biotechnology Program, Catholic University of Brasilia, Brasília 71966-700, Brazil

**Keywords:** large bone defect, bone tissue engineering, CS-based bone scaffold, P28 peptide, VEGF

## Abstract

Repairing large bone defects remains a significant clinical challenge due to the limitations of current treatments, including infection risk, donor site morbidity, and insufficient vascularization. The autograft is still the gold standard for large bone defects. In this study, we developed chitosan-based (CS-based) scaffolds, incorporating with hydroxyapatite (HAp) and fluorapatite (FAp) ceramics, fabricated by UV crosslinking and freeze-drying, and loaded with P28 peptide, alone or in combination with vascular endothelial growth factor (VEGF), to evaluate the effect of dual bioactive factor delivery. We hypothesized that CS-based scaffolds would optimize ceramic composition and co-delivery of P28 and VEGF, and can enhance early-stage osteogenic differentiation and support bone regeneration. The CS-based scaffolds were characterized by their physicochemical properties, including swelling behavior, mechanical strength, porosity, and in vitro degradation. Biological evaluations were performed including cell proliferation assays, ALP activity, ARS staining, and RT-qPCR, to assess osteogenic differentiation. The results showed that the scaffolds had high porosity, excellent swelling behavior, and degraded within 8 weeks. Dual delivery of P28 and VEGF significantly enhanced early osteogenic markers, indicating a complementary effect. These findings demonstrated that CS-based scaffolds with an optimized ceramic ratio and bioactive factor incorporation have the potential to facilitate bone regeneration.

## 1. Introduction

Bone remodeling is a continuous, dynamic process throughout life, regulated by the balance between osteoclastic resorption and osteoblastic deposition. Bone healing within a limited defect size can typically be achieved through this intrinsic remodeling mechanism [1]. However, large bone defects exceed the body’s natural regenerative capacity and therefore require medical intervention to facilitate effective bone regeneration [2]. Traditional treatments for bone defects include autografts, allografts, and xenografts, each with their own merits and limitations [3]. The gold standard for treatment is still the autograft in bone defect treatment due to its non-immunogenic character and its histocompatibility [4]. The global incidence of critical-size bone defects has been significantly increasing due to an aging population, trauma, fracture, and infection [5,6]. To address large bone defects, bone tissue engineering (BTE) has made remarkable advances in recent years [7]. The ideal bone scaffold requires mimicking the natural bone extracellular matrix (ECM), presenting favorable mechanical strength, biocompatibility, biodegradability, high porosity, and osteogenic differentiation properties. It is increasingly emerging as a promising alternative therapy for large bone defect treatment [8,9].

Chitosan/hydroxyapatite (CS/HAp) has been widely applied in BTE due to its excellent biological characteristics, minimal inflammatory reaction, osteoconductive properties, and biodegradability [10]. CS is a linear natural polymer derived from crustaceans, insects, and certain fungi, though a deacetylation reaction [11]. This compound has drawn attention as it can promote cell adhesion, and has good wettability and antimicrobial properties [12]. The main drawback of a pure CS graft is poor mechanical strength and low porosity. Thus, it is not the perfect candidate for bone grafts, while HAp, the main inorganic component of bone, can enhance the mechanical strength of a graft [13,14,15]. Moreover, HAp has demonstrated osteoconductive properties and integrates into bone without eliciting an immune reaction. HAp is a porous bio-ceramic that offers sufficient porosity for new blood vessel regeneration and cell attachment [16,17]. Fluorapatite (FAp) is prepared when OH^−^ groups in HAp are replaced with F^−^. FAp has been shown to enhance osseointegration in vitro [18].

However, BTE grafts still cannot meet revascularization needs in orthopedic medicine applications. To enhance the vascularization of BTE grafts, it is necessary to develop strategies such as cell sheet technology, added growth factors, or cell co-culture [19]. Vascular endothelial growth factor (VEGF) and bone morphogenetic protein 2 (BMP–2) are important mediators of angiogenesis and osteogenesis, respectively. VEGF also affects the processes of intramembranous ossification and endochondral ossification, except for angiogenesis [20,21]. During these processes, it is involved in various bone regeneration phases, including osteoclast recruitment, osteoblast differentiation, and chondrocyte differentiation [22]. After sufficient blood vessels have been constructed, the new bone is formed by clusters of osteocytes cells; the vessels also supply nutrition and oxygen for cell growth [23]. BMP–2, a member of the transforming growth factor–β superfamily (TGF–β), has exhibited excellent bone induction activity. Zhang et al. reported that the silk scaffold loaded with VEGF and BMP–2 was more effective during bone regeneration [24]. In fact, several studies reported that a dual-factor loading system (BMP–2/VEGF) was more efficient for vascularization and osteogenesis than one growth factor alone [25,26]. However, BMP–2 still poses some drawbacks, such as short half-life, high production cost, urinary retention, and risk of ectopic bone formation [27,28]. P28 peptide, derived from BMP–2, possesses two distinct structural elements: phosphorylated serine and a short block of seven repeated aspartic acids. P28 confers higher affinity for bone or HAp-based grafts, and also promotes the nucleation of apatite crystallization and mineralization [29,30].

Based on previous work, we successfully prepared a porous hybrid CS/HAp/FAp scaffold loaded with P28 peptide with osteoinductive properties [31]. In this study, it is demonstrated that loading VEGF/P28 onto the scaffold can be an effective strategy for inducing early osteogenic differentiation.

## 2. Results

### 2.1. Swelling Behavior

**Figure 1:**
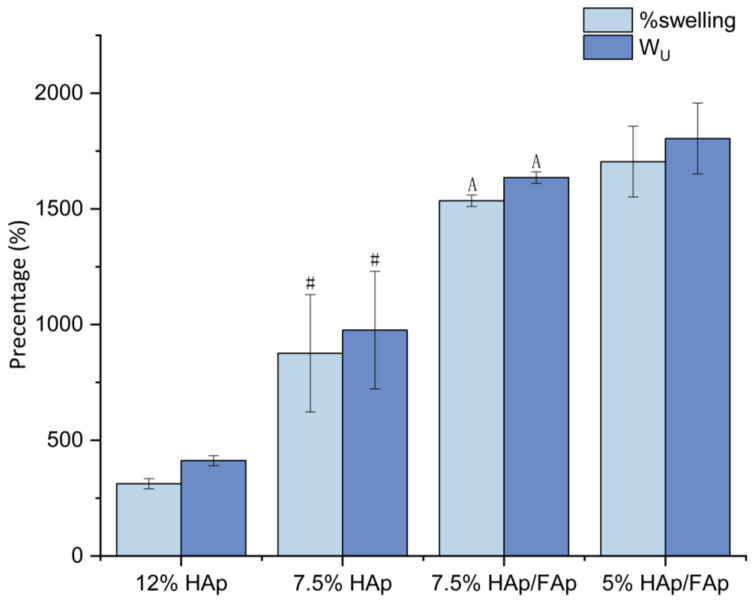
Water uptake (W_U_) and swelling percentage of CS-based scaffolds (^#^ *p* < 0.05 vs. 12% HAp, ^A^ *p* < 0.05 vs. 7.5% HAp).

The swelling behavior of the scaffolds is a key aspect of their efficacy in facilitating cell migration, attachment, and proliferation, since the pore size would increase initially and provide cell attachment sites [32,33]. The 12% HAp group exhibited the lowest % swelling and water uptake (W_u_) (*p* < 0.05) (shown in Figure 1). As the *w*/*v*% of CS paste decreased, the swelling percentage and W_u_ value increased. The addition of FAp increased the swelling of the scaffold network, which can occur in order to provide an enhanced environment for promoting cell attachment [34].

All scaffolds exhibited high PBS absorption capacity, from 300% to 1800%. The high capacity of these scaffolds could be ascribed to the existence of the hydrophilic functional groups of both CS and ceramics such as carboxyl, amino, and hydroxyl groups [25].

**Figure 2:**
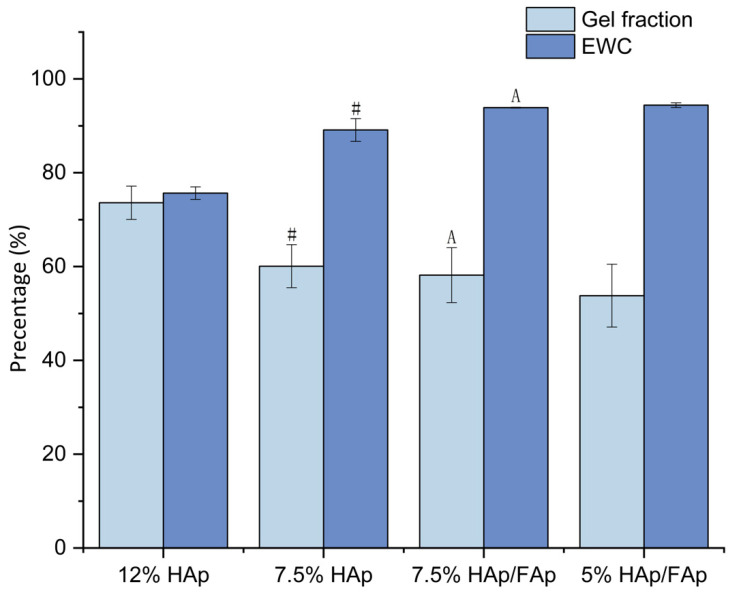
Gel fraction (GF) and equilibrium water content (EWC) of CS-based scaffolds (^#^ *p* < 0.05 vs. 12% HAp, ^A^ *p* < 0.05 vs. 7.5% HAp).

The gel fraction (GF) is an effective measurement for assessing the degree of crosslinking [35]. A higher GF indicates that a higher degree of crosslinking has occurred, which leads to a reduction in the swelling capability of the scaffolds. The crosslinking degree also affects the scaffolds’ properties, such as mechanical strength and degradation rate. GF was found to decrease with an increasing proportion of FAp, with the 5% HAp/FAp scaffold exhibiting the lowest GF at 53.79% (as shown in Figure 2). This suggested that FAp may interfere with the crosslinking process or reduce the crosslink density of the composite [34].

### 2.2. Mechanical Strength

**Figure 3:**
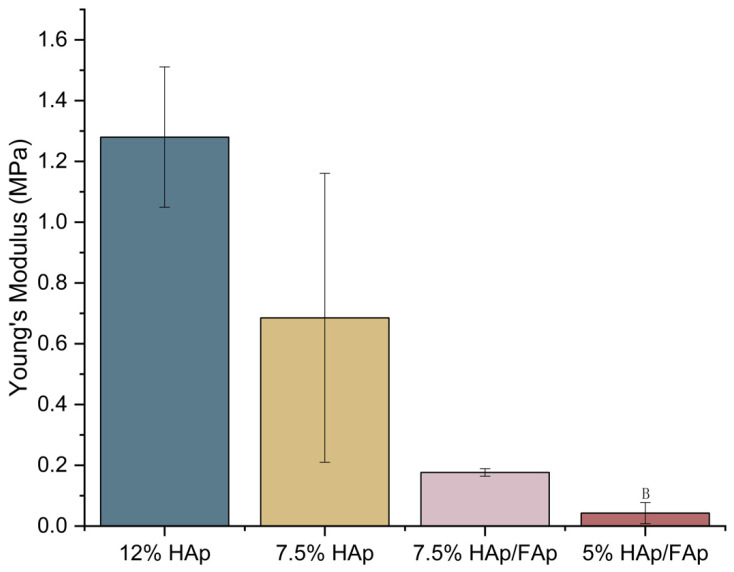
Mechanical strength of CS-based scaffolds (^B^ *p* < 0.05 vs. 7.5% HAp/FAp).

Adequate mechanical strength is an essential element of a bone scaffold since it offers mechanical loading to cells at the early stage of bone regeneration [36]. The effect of *w*/*v*% of CS and the ratio of HAp/FAp on the mechanical properties of the CS-based scaffold were analyzed (shown in Figure 3). The 12% HAp scaffold exhibited the highest mechanical strength among all scaffolds. It was observed that the compressive modulus of 7.5% scaffolds decreased after the addition of FAp, indicating that the addition of FAp decreased the formation of crosslinking chains and lowered the compressive strength of the scaffold. Moreover, the mechanical strength of the scaffolds significantly decreased as *w*/*v*% of CS paste decreased (*p* < 0.05).

### 2.3. Porosity

**Figure 4:**
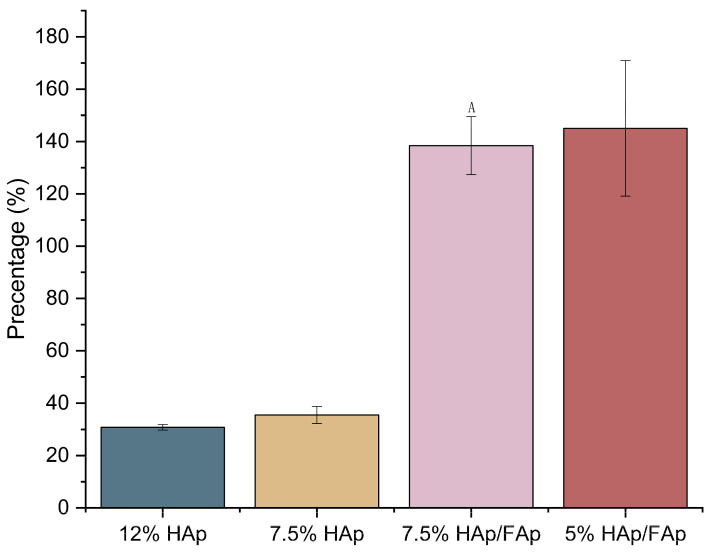
Porosity of porous scaffold (^A^ *p* < 0.05 vs. 7.5% HAp).

Porosity can directly affect the mechanical strength of the scaffold. In addition, bone regeneration relies in part on the porous structure of bone scaffolds because this characteristic facilitates angiogenesis, transporting oxygen, nutrients, and waste for cells, as well as cell migration, proliferation, and attachment [37,38]. The porosity of scaffolds significantly increased after FAp was added (*p* < 0.05). Furthermore, with a decreased *w*/*v*% of CS paste, the porosity of the scaffolds was significantly increased (shown in Figure 4). The 12% HAp scaffold had the lowest porosity among the groups. However, the addition of FAp generally reduces the GF slightly, indicating a relationship between crosslinking efficiency and water retention capability. Thus, the combination of HAp and FAp is a potentially promising scaffold as it tends to balance both GF and EWC, making it a better composite for applications requiring both high crosslinking and water retention. Additionally, the lower *w*/*v*% of CS paste contributed to a decrease in both GF and EWC. The porosity exceeding 100% was caused by the scaffolds absorbing the liquid into its structure, which resulted in overestimation of the displaced liquid volume [39].

### 2.4. In Vitro Degradation Test

**Figure 5:**
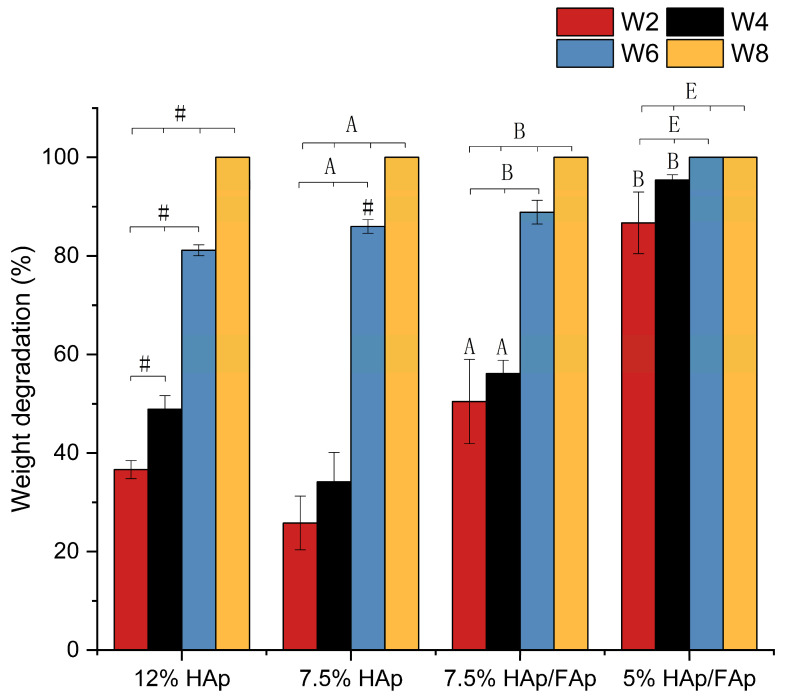
Scaffolds weight degradation percentage in W_2_, W_4_, W_6_, and W_8_ time points (^#^ *p* < 0.05 vs. 12% HAp, ^A^ *p* < 0.05 vs. 7.5% HAp, ^B^ *p* < 0.05 vs. 7.5% HAp/FAp, ^E^ *p* < 0.05 vs. 5% HAp/FAp).

The weight retention results reflect the scaffold’s degradation profile, providing insights into its structural stability over the studied period. The degradation rate of scaffolds was observed over a period of 8 weeks. Figure 5 shows that the lowest degradation rate over the 8 weeks period was seen with 12% HAp scaffold and 7.5% HAp scaffold. This indicated that these scaffolds had superior structural integrity and are highly resistant to degradation, making them suitable for applications requiring long-term stability [40,41]. However, the 5% HAp/FAp scaffold degraded quickly, losing over 80% after 2 weeks (Figure 5).

### 2.5. Cell Proliferation

**Figure 6:**
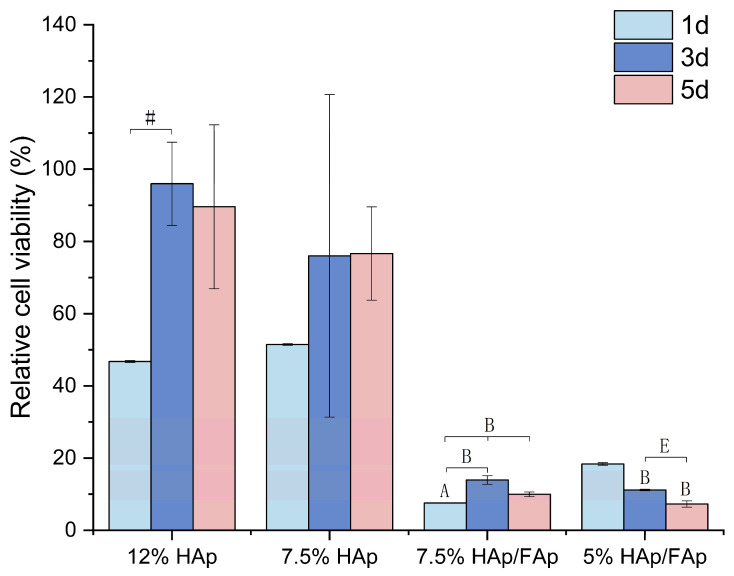
CCK–8 assay was used for cells proliferation evaluation (^#^ *p* < 0.05 vs. 12% HAp, ^A^ *p* < 0.05 vs. 7.5% HAp, ^B^ *p* < 0.05 vs. 7.5% HAp/FAp,^E^ *p* < 0.05 vs. 5% HAp/FAp).

C2C12 cells were seeded at the same initial density and cultured with the scaffolds for 1, 3, and 5 days to evaluate cell proliferation using the CCK–8 assay. As shown in Figure 6, a comparison of cell proliferation between cells alone and those co-cultured with the scaffolds showed that C2C12 cells proliferated with the scaffolds (12% HAp and 7.5% HAp) and displayed a time-dependent behavior, while the viability of cells increased rapidly along with culture time. In addition, HAp composite scaffolds showed better proliferation compared to the HAp/FAp composite scaffolds. However, the variation in *w*/*v*% of CS paste had a slight negative effect on cell proliferation.

### 2.6. Alizarin Red S Stain

**Figure 7:**
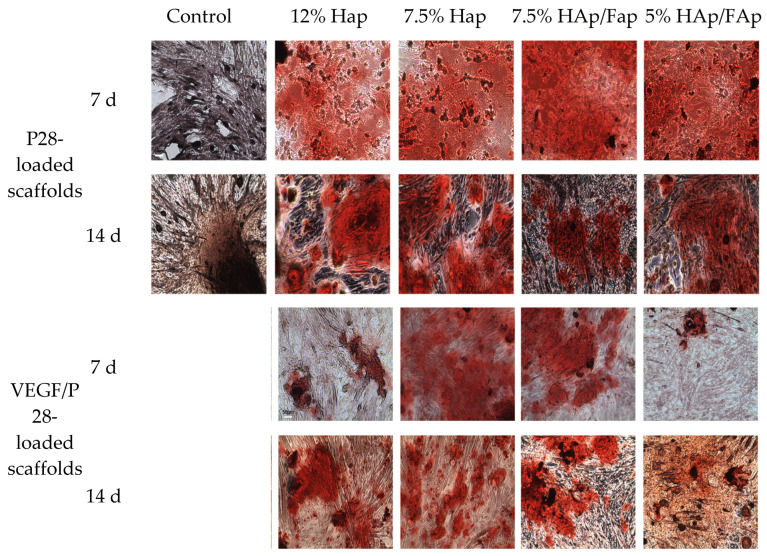
Alizarin Red S staining for C2C12 cells after culturing with scaffolds for 7 and 14 days (the **upper section** showed the ARS staining results of P28-loaded scaffolds (the data of P28-loaded scaffolds in this figure were previously published in our earlier work [31], and are reused here for comparison), while the **lower section** showed the ARS staining results of VEGF/P28-loaded scaffolds; scale bar= 50 μm).

ARS was used to analyze the presence of ECM mineralization. Calcium nodule deposition is a biomarker for late-stage osteogenic differentiation [42,43]. In this study, the differentiation of C2C12 co-cultured with VEGF/P28-loaded scaffolds was assessed after 7 and 14 days. It can be concluded that the VEGF/P28-loaded CS/ceramic scaffolds provide an ideal environment for promoted osteogenesis of C2C12 cells. The VEGF/P28-loaded scaffolds exhibited stronger ARS staining than the control group, indicating a greater amount of mineralized matrix (shown in Figure 7). Moreover, the VEGF/P28-loaded 7.5% HAp group exhibited significantly more mineralized nodules than the VEGF/P28-loaded 12% HAp group. However, no significant difference was detected between the VEGF/P28-loaded 7.5% HAp group and the VEGF/P28-loaded 7.5% HAp/FAp. The results of ARS staining displayed that the calcium nodules were almost entirely covered in C2C12 co-cultured the P28-loaded scaffolds, which were darker in color and larger in size. However, the 7.5% HAp group exhibited similar results when VEGF/P28- or only P28-loaded. Moreover, the VEGF/P28-loaded 5% HAp/FAp showed significantly smaller calcium nodules than P28-loaded 5% scaffold.

**Figure 8:**
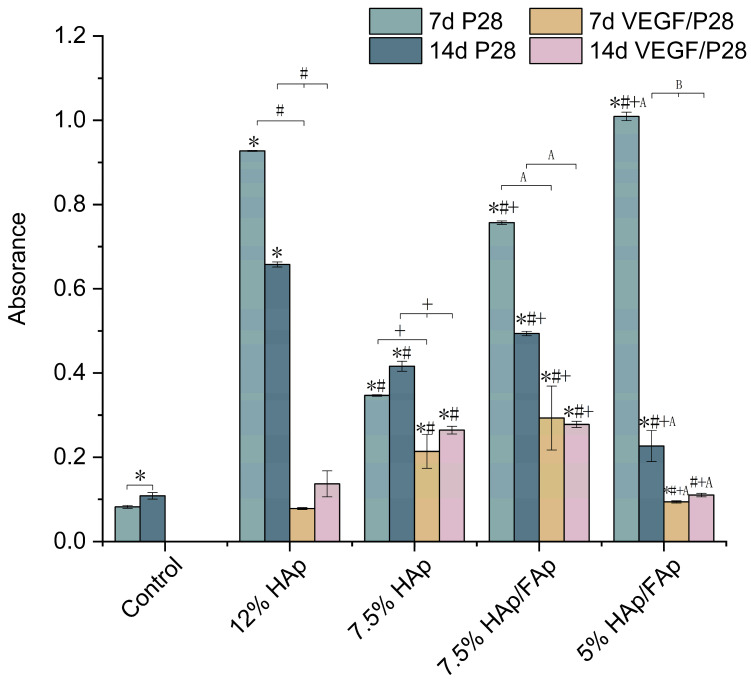
The quantification of ARS for different formulations of scaffolds and peptide-loaded scaffolds at 7 and 14 days (the data for P28-loaded scaffolds in this figure were previously published in our earlier work [31], and are reused here for comparison.) (* *p* < 0.05 vs. control, ^#^ *p* < 0.05 vs. 12% HAp, ^+^ *p* < 0.05 vs. 7.5% HAp, ^A^ *p* < 0.05 vs. 7.5% HAp/FAp, ^B^ *p* < 0.05 vs. 5% HAp/FAp).

Further quantitative analysis of ARS staining demonstrated that the OD value in the VEGF/P28-loaded group was significantly higher than that in the control group (*p* < 0.05). Compared with the 7 d, the OD values of calcium nodule deposition significantly increased after 14 d in all VEGF/P28-loaded scaffolds except for 7.5% HAp/FAp. These results demonstrated that the VEGF/P28-loaded scaffolds had a proliferative effect at a later stage of osteogenic differentiation. Furthermore, the OD of VEGF/P28-loaded 7.5% HAp was significantly higher than that of VEGF/P28-loaded 12% Hap, in line with colorimetric quantification results. However, the P28-loaded scaffolds exhibited greater efficacy than VEGF/P28-loaded scaffolds (Figure 8), as illustrated by the stronger red stain compared to VEGF/P28 scaffolds.

### 2.7. Alkaline Phosphatase Activity

**Figure 9:**
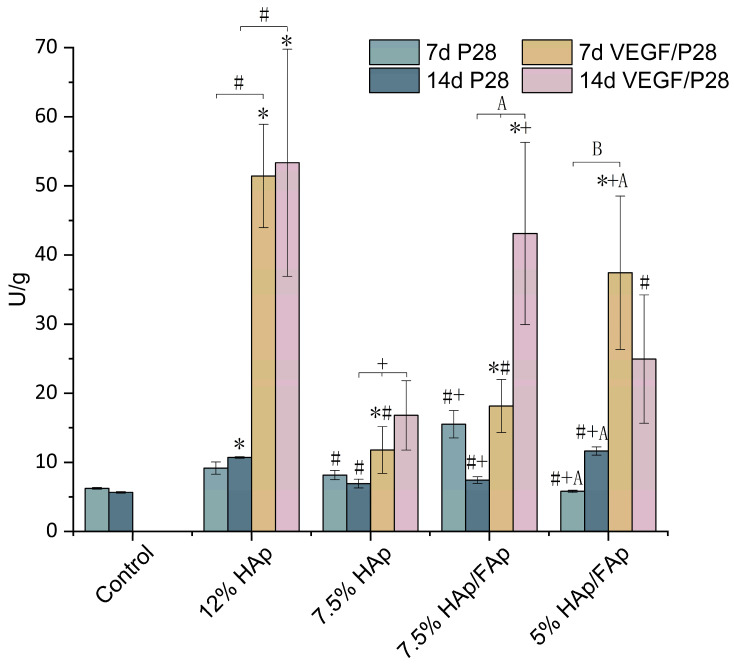
Quantification of ALP with different formulations of P28-loaded scaffolds and VEGF/P28-loaded scaffolds at 7 and 14 days (the data for P28-loaded scaffolds in this figure were previously published in our earlier work [31], and are reused here for comparison) (* *p* < 0.05 vs. control, ^#^ *p* < 0.05 vs. 12% HAp, ^+^ *p* < 0.05 vs. 7.5% HAp, ^A^ *p* < 0.05 vs. 7.5% HAp/FAp, ^B^ *p* < 0.05 vs. 5% HAp/FAp).

Cellular alkaline phosphatase (ALP) activity is an early characteristic parameter of osteoblast differentiation [10,44]. ALP activity of cells cultured for 7 d and 14 d was examined. As presented in Figure 9, the ALP activity of C2C12 cells increased with incubation time, except for the VEGF/P28-loaded 5% HAp/FAp group. The ALP activities of the VEGF/P28-loaded 12% HAp group were significantly higher than the VEGF/P28-loaded 7.5% HAp group (*p* < 0.05). On day 7, the ALP activity of C2C12 cells cultured in the presence of VEGF/P28-loaded scaffold groups was significantly higher than in the control. Conversely, the ALP activity of the VEGF/P28-loaded 7.5% HAp group was similar to the control group on day 14. Furthermore, the VEGF/P28-loaded scaffolds showed significantly higher ALP activity than P28 scaffolds alone. For VEGF/P28-loaded scaffolds, the ALP activities increased with time; in particular, the 7.5% HAp/FAp scaffolds significantly increased by 14 d (*p* < 0.05).

### 2.8. Gene-Expression Analysis

**Figure 10:**
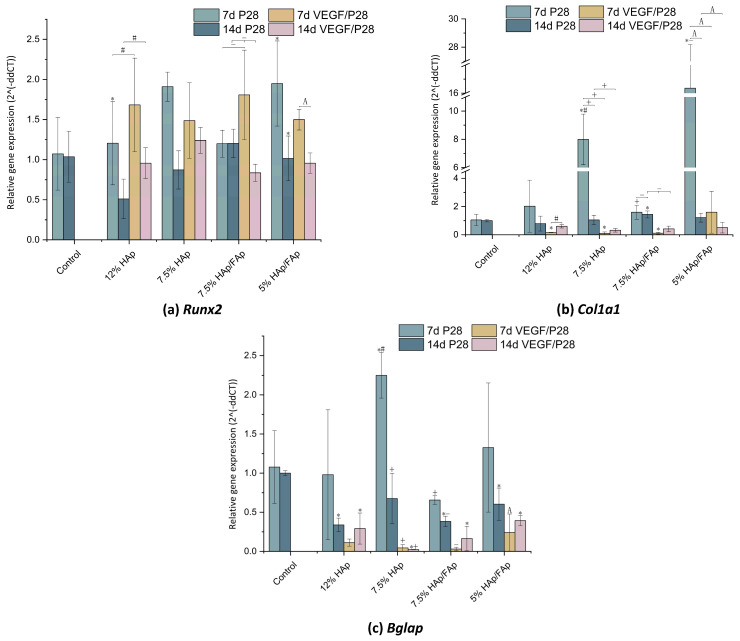
Relative levels of bone-associated RNAs for *Runx2* (**a**), *Col1a1* (**b**), and *Bglap* (**c**) from C2C12 cells seeded onto porous scaffolds substrates and incubated for 7 and 14 days in the presence or absence of the peptide (the data for P28-loaded scaffolds in this figure were previously published in our earlier work [31], and are reused here for comparison.) (* *p* < 0.05 vs. control, ^#^ *p* < 0.05 vs. 12% HAp, ^+^ *p* < 0.05 vs. 7.5% HAp, ^−^ *p* < 0.05 vs. 7.5% HAp/FAp, ^A^ *p* < 0.05 vs. 5% HAp/FAp).

The expression of osteogenesis-differentiation genes, *Runx2*, *Col1a1*, and *Bglap*, was measured through RT-qPCR, and the results are plotted in Figure 10. *Runx2* is a crucial transcription factor in osteoblast differentiation [42]. Compared to P28-loaded 12% HAp scaffold, the *Runx2* gene expression of the VEGF/P28-loaded 12% HAp was significantly upregulated at 7 d (*p* < 0.05) (Figure 10a). The same was true for the 7.5% HAp/FAp scaffold. On the other hand, both the 7.5% HAp and 5% HAp/FAp showed lower (though not statistically significant) expression of *Runx2* in the VEGF/P28-loaded scaffolds compared to P28-loaded. In all cases, the expression returned to baseline at 14d. In all scaffold formulations, expression of *Col1a1* and *Bglap* was reduced in the VEGF/P28-loaded scaffolds compared to the P28-loaded ones (Figure 10b,c). In most cases, expression was also reduced compared to the control. Differently from previous profiles, in some cases, the expression levels of *Col1a1* and *Bglap* showed a modest increase at 14 d for the VEGF/P28-loaded scaffolds, though it is important to point out that this is in comparison to strongly reduced expression levels at 7 d.

## 3. Discussion

This study investigated the influence of the different *w*/*v*% of CS paste and HAp/FAp ratios loaded with bioactive factors on the physical characteristics of scaffolds and their osteogenic differentiation ability following fabrication using UV crosslinking and freeze drying.

The selected process used here was lyophilization, which preserves the integrity of the three-dimensional scaffold structure and forms porous scaffolds through the sublimation of water at low pressure under vacuum [45]. An important feature of bone scaffolds is controlled swelling behavior and high porosity to facilitate bone regeneration [46]. Appropriate swelling behavior is essential to increase the surface area of the bone-scaffold-promoting cell attachment and proliferation, and also for nutrient absorption and waste transfer [47]. The swelling behaviors of these scaffolds indicate that the addition of FAp and the lower concentration of CS paste significantly enhance the % swelling and W_U_. Conversely, the high swelling capacity of the scaffolds exhibited slightly lower GF, suggesting a decreased degree in cross-linking and the formation of a more porous network. In the swelling study, the 7.5% HAp/FAp scaffold exhibited a favorable balance of high W_u_ and moderate GF, suggesting that it provides sufficient hydration for cell attachment while maintaining acceptable structural stability.

High porosity and an interconnected pore structure are essential for BTE scaffolds [42]. High porosity offers the space to promote cell attachment, proliferation and differentiation, and facilitate the invasion of blood vessels to transport nutrients and oxygen to support bone regeneration [48]. The porosity of the scaffolds provides ideal interconnectivity to ensure good proliferation of cells [49]. It was reported that the scaffold porosity should be at least 75% in BTE [50]. As the results indicate (Figure 4), some scaffolds (7.5%HAp/HAp and 5% HAp/FAp) had higher porosity (over 75%), which indicated that FAp contributes to increased scaffold porosity. This was likely due to FAp having weaker interactions with CS matrix due to the lower hydrophilicity and caused the formation of larger or more numerous pores formed in scaffolds as reported in the literature [51]. However, the HAp scaffolds had lower porosity, which is not suitable for cell infiltration and attachment, as well as osteogenesis [46]. On the other hand, HAp exhibited a complementary effect with FAp in increasing the porosity of scaffolds, as the irregularly shaped ceramic particles facilitated the formation of voids within the material [52,53]. Furthermore, the varying *w*/*v*% of CS paste also affected the scaffolds’ porosity, with the scaffolds’ porosity tending to increase as the *w*/*v*% of CS paste decreased. The lower *w*/*v*% of CS paste reduced viscosity, which increased protonation, decreasing chain entanglement, and disrupting hydrogen bonding. The lower viscosity enhances bubble expansion, facilitates solvent removal, and allows for better phase separation, leading to larger and more interconnected pores in the scaffold [54,55]. Among all formulations, the 7.5% HAp/FAp scaffold demonstrated the most desirable porosity (>75%), offering interconnected pore networks that support cell infiltration, vascularization, and bone tissue ingrowth, while still maintaining structural coherence, making it a promising candidate for BTE applications.

CS-based scaffolds have been shown to have significant degradation behavior in several studies [56,57]. Moreover, the biodegradable material can reduce the risk of bone infection due to it is gradual absorption and replacement by new bone tissue. Biodegradability of the porous scaffolds was estimated by studying the weight of scaffolds at different time points intervals the weight of scaffolds. All scaffolds exhibited progressive weight reduction over time, with significant decreases observed between each time point (W_2_, W_4_, W_6_ and W_8_). After freeze-drying, the scaffolds degraded completely within 8 weeks. However, the 5% HAp/FAp scaffold degraded too quickly, which is not compatible with the bone regeneration process since bone fractures typically heal within 6–8 weeks [58]. In addition, the incorporation of FAp contributed to more rapid scaffold degradation, resulting in a lower efficacy of bonding with the CS matrix [59]. The lower concentration of CS paste resulted in weaker molecular interactions due to the stronger protonation effect [60].

The mechanical properties of the scaffold play a crucial role in BTE, as the scaffold must withstand physiological loads while providing a supportive environment for cell growth during bone regeneration [61,62]. The 7.5% HAp scaffold had better mechanical strength due to the high hydrophilicity of HAp, which enhanced the interaction with CS matrix [63]. The lower *w*/*v*% of CS paste induced a lower-density polymer network (lower GF) and decreased the compressive strength of the scaffold in line with findings of other researchers [64]. The 7.5% HAp scaffold exhibited the most suitable degradation rate and mechanical strength for BTE. Therefore, it is essential to analyze the scaffolds’ properties to confirm a balance between mechanical strength and porosity.

CCK–8 was used to assess cell proliferation at 1, 3 and 5 days in the presence of scaffolds. In this study, some groups (12% HAp and 7.5% HAp) showed increased absorbance, which was consistent with progressive cell proliferation [65,66]. The ceramic provides the roughness features for cells adhesion [67]. However, the 7.5% and 5% HAp/FAp presented cytotoxicity at the beginning of the co-culture stage. In addition to potential ionic effects, in the physical presence of degraded 7.5% and 5% HAp/FAp scaffolds, fragments adhered to the cell layer may also cause reduced cell proliferation, as these fragments can partially cover the cells and restrict nutrient and oxygen diffusion at the local interface [39]. Despite this, the cell proliferation still increased at a later co-culture stage with 5% HAp/FAp, indicating that the scaffolds promoted the cell proliferation process [68]. Although CCK–8 provided quantitative evidence of cell proliferation, a limitation of this study was the absence of microscopic images, including different stages of swelling and degradation to verify cell attachment and proliferation on the scaffold, thus directly verifying these observations. SEM analysis of swollen scaffolds or fluorescence microscopy would have further confirmed cell attachment and interpenetration within the scaffold structures. Therefore, future work will investigate microscopic imaging of swollen scaffolds to complement the quantitative assays and provide direct morphological evidence of scaffold cell interactions.

Alizarin Red S (ARS) staining was used to assess the osteogenic differentiation of C2C12 cells co-cultured with various scaffolds loaded with either VEGF/P28 or P28 alone. As a sensitive late-stage indicator of osteogenesis, ARS specifically detects matrix mineralization [69,70]. In Figure 7, it was observed that all scaffolds exhibited good osteogenic potential and increased progressive mineralization over time. The control group showed minimal mineralization at 7 and 14 d, which indicated that cells did not sufficiently differentiate in the absence of the bioactive factor-loaded scaffolds. The most effective group was 7.5% HAp/FAp scaffold among VEGF/P28-loaded groups, which indicated that the combination of HAp and FAp had a complementary effect on enhancing calcium deposition, as previously reported [71]. However, the lower *w*/*v*% of CS paste may adversely affect CS’ structural properties or mineralization dynamics. Scaffolds loaded with P28 exhibited significantly higher ARS staining values than those loaded with VEGF/P28 (*p* < 0.05). P28 directly stimulated osteogenic differentiation and mineral deposition, contributing to the increased ARS values observed in P28-loaded scaffolds [72]. However, the incorporation of VEGF may introduce competition or interference in signaling pathways, redirecting cellular activity toward vascularization rather than mineralization. The addition of VEGF reduced the osteogenic differentiation efficacy of P28 by altering its concentration gradient or bioavailability in vitro. Grosso et al. (2017) reported a loss of VEGF impaired osteoblast differentiation and bone deposition; however, overexpression also led to excessive bone resorption and likewise caused bone loss [73].

To further investigate the osteogenic differentiation in the C2C12 cells co-cultured with various bioactive factor-loaded scaffolds, ALP activity was assessed by the fluorometric method, as an early marker of osteoblast differentiation [10,43]. The control groups showed the lowest ALP activity, while the VEGF/P28-loaded 12% HAp scaffold exhibited the highest ALP activity among the groups. Furthermore, the 7.5% HAp/FAp scaffold demonstrated ALP activity exceeding that of 7.5% HAp scaffold which emphasized the complementary effect of HAp and FAp, also reflected in ALP activity corresponding with ARS results. Fluoride ions strengthen the interaction capacity of scaffolds with cells, which activates specific signaling pathways for enhancing osteoblast differentiation [74]. Compared with the VEGF/P28-loaded 12% HAp scaffold, the VEGF/P28-loaded 7.5% HAp scaffold exhibited a reduction in ALP activity, indicating that an excessively lower *w*/*v*% of CS paste may inhibit cellular activity and disrupt the differentiation-to-mineralization cascade. The results demonstrated that the addition of VEGF significantly influenced ALP activity, suggesting that VEGF may amplify differentiation at early osteogenic stages [75]. Angiogenesis and osteogenesis are closely coupled processes, but excessive angiogenesis may outcompete osteogenesis for cellular resources, particularly at later stages. In VEGF/P28-loaded scaffolds, the prolonged stimulation of angiogenesis may suppress mineralization. The controlled release strategy for VEGF and P28, where VEGF is delivered early and P28 persists for later stages, could help optimize both angiogenesis and mineralization for BTE applications [76].

The expression levels of *Runx2*, *Col1a1*, and *Bglap* (osteocalcin) were detected as indicators for the osteogenic differentiation of C2C12 cells that were co-cultured with various bioactive factor-loaded scaffolds at 7 and 14 days [77]. C2C12 cells have the ability to undergo osteoblastic differentiation under the appropriate conditions. Sharma et al. reported that VEGF alone failed to promote osteogenic activity both in vitro and in vivo [78]. VEGF facilitates the bone formation by enhancing BMSCs recruitment at the early stage [79]. BMP-2 promoted osteogenic differentiation gene upregulation by inducing the *Runx2* expression via the Smad signaling pathway. *Runx2* is a master transcription factor for differentiation of mesenchymal precursor cells into osteoblasts [80]. The *Runx2* gene was downregulated over time, reflected the early osteogenic differentiation stage and mineralization stage [81]. As presented in Figure 10a, the *Runx2* was observed to be upregulated expression in response to cell co-culture with both the P28 and VEGF/P28-loaded scaffolds. The upregulation of *Runx2* demonstrated that both bioactive factors-loaded scaffolds could facilitate the differentiation of C2C12 cells. *Col1a1* is an ECM marker in cartilage tissue and downregulates in the mineralization phase. Compared to the P28 alone groups, the *Col1a1* expression of VEGF/P28 groups was downregulated. It was observed that VEGF/P28 groups may remain in the early stage of osteogenic differentiation, as also indicated by the ARS and ALP results. The VEGF/P28 scaffold performed better at the early stage of osteogenic differentiation. VEGF primarily promotes angiogenesis while limiting its direct osteoinductive effects on C2C12 cells, thus requiring a strong osteogenic inducer such as BMP-2. The P28-loaded 5% HAp/FAp scaffold showed the highest *Col1a1* expression level at 7 d, further suggesting that HAp and FAp had a complementary effect on early osteogenic differentiation. Patel et al. reported that the VEGF/BMP-2 delivery induced significantly increased bone formation compare with BMP-2 or VEGF alone at early stages (4 weeks); however, there was no significant difference between VEGF/BMP-2 and BMP-2 alone at late stages (12 weeks) [82]. *Bglap* expression can reflect the extent of bone formation, and is secreted by terminally differentiated osteoblasts. It is considered an osteogenic differentiation marker in the late stage and regulates bone calcium metabolism [83,84]. The ARS results for VEGF/P28-loaded scaffolds showed a decrease compared with P28-loaded scaffolds, following the same trend observed in the expression levels of *Col1a1* and *Bglap,* suggesting a decline in matrix production or mineralization activity at later stages. Nevertheless, despite the lower gene expression, ARS results confirmed the presence of calcium matrix deposition, indicating that mineralization was still taking place, albeit at a possibly reduced rate. We hypothesized that this apparent disconnect highlights a temporal mismatch between gene expression and phenotypic mineral deposition, which may result from the angiogenic priming effect of VEGF, and warrants further investigation at additional time points. The P28-loaded 7.5% HAp scaffold presented markedly higher levels of gene expression among all the scaffolds at 7 d, which supports the HAp in promoting osteoblast differentiation and matrix mineralization. It has also been observed that VEGF inhibited osteogenesis in vitro by supplementation or overexpression, and VEGF is a potent inhibitor of BMP-2 expression in MSCs. P28-loaded scaffolds were more effective than VEGF/P28-loaded scaffolds at late-stage mineralization due to VEGF interfering with the P28 signaling pathway, which limited the osteogenic differentiation process at the late stage [85]. Among the scaffolds, P28-loaded 7.5% HAp/FAp showed superior performance at early stages of gene expression, making it the most promising formulation for complete osteogenic differentiation. Although the release kinetics of P28 and VEGF were not evaluated, the biological results demonstrated that VEGF contributed to osteogenic differentiation at the early stages, indicating that the incorporated bioactive factors retained bioactivity. Nevertheless, the absence of release data represents a limitation, as the timing and duration of bioactive factor availability are critical for coordinating osteogenic and angiogenic processes. A comprehensive evaluation of P28 and VEGF release kinetics would allow a more accurate correlation between scaffold composition and the observed cellular outcomes. Therefore, future studies will prioritize the systematic characterization of P28/VEGF release behavior to optimize delivery strategies and maximize scaffold performance.

## 4. Materials and Methods

Poly(ethylene glycol) dimethacrylate (PEGDMA, MW 600) was acquired from PolySciences Inc (Polysciences Europe GmbH, Hirschberg an der Bergstrasse, Germany). Sodium bicarbonate (NaHCO_3_) was supplied by Fisher Scientific (Dublin, Ireland). The osteogenic peptide ({pS}DDDDDDDKIPKASSVPTELSAISTLYL, P28 peptide) was synthesized by the Pepmic (Suzhou, China).

Mouse VEGF–165 recombinant protein and RT–qPCR-related reagents were purchased from Thermo Fisher Scientific (Dublin, Ireland). The alkaline phosphatase assay kit and cell lysis buffer were obtained from Beyotime Institute of Biotechnology (Shanghai, China). The C2C12 murine myoblast cell line was purchased from the European Collection of Cell Cultures (ECACC, Salisbury, UK). The other reagents used in the experiments were purchased from Sigma–Aldrich (Wicklow, Ireland).

### 4.1. Scaffold Preparation

The CS/HAp/FAp scaffolds (shown in Table 1) were prepared by UV irradiation. For scaffold preparation, various ratios of HAp and FAp were dissolved in distilled water and mixed for 1 h. At the same time, 5%, 7.5% and 12% *w*/*v* CS paste was prepared with 1% acetic acid (AA) solution in distilled water which was left on bench for 1 h. Subsequently, the CS paste was immersed in 0.1 M sodium bicarbonate for neutralization. The CS paste was thoroughly mixed with ceramic solution until a homogeneous paste was obtained. Then, PEGDMA 600 and 0.1% *w*/*v* benzophenone solution in ethanol was added to the homogenous paste and mixed well. To initiate the photocrosslinking reaction, the paste was placed into disk shaped silicon molds and placed into UV chamber (Dr. Gröbel UV-Electronik GmbH, Ettlingen, Germany). After 40 min of UV curing, the scaffolds were placed in −80 °C freezer overnight. Finally, the scaffolds were freeze-dried (Heto LyoLab 3000, Thermo Scientific, Loughborough, UK).

### 4.2. Swelling Behavior

The UV crosslinked and freeze-dried scaffolds (diameter: 24 mm, height: 2 mm) were weighed (W_d_) and immersed in PBS (pH: 7.4) at room temperature for 48 h to allow them to swell to equilibrium. The swollen scaffolds were reweighed (W_s_) after removal of excess surface water using filter paper. The equilibrium water content (EWC), percentage swelling, and water uptake (W_u_) were calculated using the following equations [35].

(1) $$EWC=\frac{W_{s}-W_{d}}{Ws}\;*\;100$$

(2) $$W_{u}=\frac{W_{s}-W_{d}}{W_{d}}\;*\;100$$

(3) $$\%\;swelling=\frac{W_{s}}{W_{d}}\;*\;100$$

W_d_: the weight of dried scaffold; W_s_: the weight of swollen scaffold.

The dried scaffolds were weighed (W_a_) and submerged into 1% acetic acid solution for 48 h, and then weighted the scaffolds (W_e_) after redrying. GF was used to analyze the efficacy of the crosslinking reaction.

(4) $$Gel\;fraction=\frac{W_{e}}{W_{a}\;}\;*\;100$$

W_a_: the weight of dried scaffold; W_e_: the weight of swollen scaffold.

### 4.3. Compression Test

The Lloyd LRX universal with 2.5 kN load cell was used to determine the mechanical strength of scaffolds. The scaffolds were equilibrated in phosphate-buffered saline (PBS, pH 7.4) for 1 h at room temperature before test. Triplicate tests of each batch were performed at a 0.5 mm/min rate until achieving a 60% strain. The Young’s modulus was automatically calculated by the instrument software.

### 4.4. Porosity Test

The porosity of scaffolds was assessed using the liquid displacement method. The dried scaffolds (*n* = 3) were submerged in distilled water until reaching equilibrium after 36 h. Porosity calculations were based on the difference in scaffold weight before (W_1_) and after (W_2_) water immersion, divided by the product of the scaffold’s dried volume (Vs) and the density of distilled water. Results were reported as the average plus or minus the standard deviation for three samples.

Porosity% = (W_2_ − W_1_)/ ρVs (5)

### 4.5. In-Vitro Degradation Test in Simulated Body Fluid (SBF)

The ability of porous scaffolds to degrade was evaluated by determining the weight loss percentage of scaffolds in SBF in vitro. SBF was prepared according to Kokubo et al. (2006) [86] protocol, matching human blood plasma’s ion concentrations closely, as shown in Table 2 (Na^+^, K^+^, Ca^2+^, Cl^−^, HCO_3_^−^, HPO_4_^2−^, SO_4_^2−^). SBF was buffered at pH 7.4 with tris (hydroxymethyl) aminomethane and 1 M hydrochloric acid (HCL) at 37 °C. Prior to experiments, the dried scaffolds were sterilized under UV light for 30 min, followed by weighing the scaffolds (W_0_).

The scaffolds were immersed into SBF solution totally, and incubated in a shaking incubator. The buffer solution was changed twice a week, addressing the reduction in cation levels and pH change. The scaffolds were removed at predetermined time intervals of 2, 4, 6, and 8 weeks. The scaffolds were gently rinsed twice with distilled water and dried to equilibrium. The percentage of weight loss was calculated according to the following equation:

Degradation weight percentage = (W_0_ − W_1_)/W_0_ × 100 (6)

W_0_: the initial weight of dried scaffold; W_1_: the weight of dried scaffold after immersed into SBF.

### 4.6. Cell Proliferation

The cell proliferation of different nude scaffolds was assessed by cell counting kit–8 (CCK–8) assay (Beyotime, Shanghai, China) (*n* = 3 for each group). Samples of 5 × 10^3^ cells/mL were cultured in each well of a 96-well plate. After the cells grew to 80% confluence, the sterilized scaffolds were placed on cells for 1, 3 and 5 days. The sterilized scaffolds were removed from the wells, and each well was washed twice using PBS at this time. A 100 μL CCK–8 solution with 10% volume of culture medium (DMEM) was added, followed by incubation at 37 °C and 5% CO_2_ for 2 h. The results were assessed at 450 nm using a BioTek Synergy HTX Multimode Reader (Agilent, Santa Clara, CA, USA).

### 4.7. Bioactive Factors Loading

The dried scaffolds (d = 4.8 mm, h = 1.7 mm) were sterilized for 30 min in a UV chamber in advance. An amount of 5 mg P28 peptide (S[PO4]DDDDDDDKIPKASSVPTELSAISTLYL, molecular weight: 3091.20) was dissolved in 500 μL of medium and mixed well. P28-loaded scaffolds were prepared by dropping 15 μL P28 solution (10 mg/mL) onto the scaffolds under sterile conditions. Finally, P28-loaded scaffolds were stored at −20 °C for later use.

The 50 μg VEGF was dissolved in 100 μL medium, and 2 μL VEGF solution was dropped on the scaffolds. The P28/VEGF-loaded scaffolds were stored at −20 °C for later use.

### 4.8. Cell Seeding for Osteogenic Markers Analysis and Gene Expression

In the cell culture assay, the C2C12 cells (4 × 10^4^ cells/well) were seeded on a 12-well plate and incubated in Dulbecco’s Modified Eagle’s Medium–high glucose (DMEM) medium containing 10% fetal bovine serum (FBS), 1% penicillin-streptomycin and 1% L-Glutamine at 37 °C in a 5% CO_2_ incubator.

### 4.9. Alizarin Red S Staining (ARS)

Alizarin red S staining was used to determine the calcium nodule formation. The cells were cultured with scaffolds for 7 d and 14 d, and then fixed with 4% paraformaldehyde for 30 min. Subsequently, the paraformaldehyde was removed, and samples were washed with PBS twice. Next, 40 mM ARS solution was used to stain the samples for 15 min at room temperature. Afterwards, the stained samples were washed three times with distilled water, and images were captured with a digital camera and microscope.

The quantification of calcium nodules was assessed after the stained samples were extracted by 10% acetic acid. The optical density of samples was determined spectrophotometrically at 405 nm.

### 4.10. Alkaline Phosphatase (ALP) Activity

ALP was qualitatively assessed to examine early osteogenic differentiation of C2C12 cells in the presence of the scaffold. The scaffolds were cultured with cells for 7 and 14 days. At the end of incubation periods, the cells were washed three times with PBS and lysed by 1% Triton X–100 at 4 °C (Beyotime, Shanghai, China). After 30 min incubation, the colorless p-nitrophenyl phosphate (pNPP) was converted to colored p-nitrophenol by ALP. Then, the results were normalized to total intracellular protein measured with bicinchoninic acid (BCA) Protein Assay Kit.

### 4.11. Reverse Transcription Quantitative Polymerase Chain Reaction

RNA was isolated from C2C12 cells on 7 d and 14 d using Purelink RNA Mini Kit (Thermo Fisher Scientific, Waltham, MA, USA) following the manufacturer’s protocol and the RNA quantitation was assessed by Qubit fluorimeter (Thermo Fisher Scientific, Waltham, MA, USA). For reverse-transcription quantitative polymerase chain reaction (RT-qPCR), the cDNA was synthesized by High Capacity kit (Thermo Fisher Scientific, Waltham, MA, USA) with RNase inhibitor as per the instructions provided. The synthesized cDNA was stored at −20 °C for later analysis.

The expression values were determined by real time RT-qPCR with PowerUp^TM^ SYBR^TM^ Green Master Mix (Thermo Fisher Scientific, Waltham, MA, USA). Each reaction consisted of 5 μL of the master mix; 3.5 μL of molecular grade water; 0.25 μL of each primer (forward and reverse) from a 10 μM stock; and 1 μL of cDNA (template). The reaction was performed with the 7300 Real-Time PCR System (Applied Biosystems, Waltham, MA, USA), using the “standard 7300” run mode and the following settings: 1 cycle at 50 °C for 2 min; 1 cycle at 95 °C for 2 min; 45 cycles at 95 °C for 15 s, 60 °C for 15 s, and 72 °C for 1 min, followed by a dissociation stage of 95 °C for 15 s, 60 °C for 1 min, 95 °C for 15 s, and 60 °C for 15 s. *Tbp* was used as a housekeeping gene, and *Bglap*, *CoI1a1*, and *Runx2* were used as specific markers of osteoblast cells. Relative expression was determined by the 2^(−ΔΔCt) method. All the sequences of primers are shown in Table 3.

### 4.12. Statistical Analysis

The results were obtained from triplicate samples and presented as mean ± standard deviation. Statistical analysis of data was carried out by one-way analysis of variance (ANOVA) with Minitab 15.0, where *p* ≤ 0.05 was considered statistically significant.

## 5. Conclusions

Three-dimensional biodegradable CS/HAp/FAp scaffolds were investigated in this study. These novel scaffolds offer excellent swelling behavior, biocompatibility and osteogenic differentiation properties in vitro, which can be tested in bone regeneration applications in the future. Additionally, this work comprehensively evaluated the potential for the incorporation of FAp into bone scaffolds. Future research is required to investigate the regenerative capacity of Fap-incorporated bone scaffolds as a potential treatment for large bone defects. The *Col1a1* gene expression showed similar trends to the *Runx2* gene. In contrast, the VEGF/P28-loaded scaffold downregulated the *Col1a1* gene expression compared to the P28-loaded scaffold, consistent with ARS results. P28 directly stimulates osteogenic differentiation, enhancing the expression of *Col1a1* and other bone matrix-related genes. In the context of P28-loaded scaffolds, its upregulation is essential for ECM production and subsequent mineralization, making it a pivotal factor in bone tissue engineering and regenerative therapies. These findings highlight the importance of scaffold composition, controlled VEGF/P28 release, and optimized fluoride incorporation in enhancing bone regeneration. The results demonstrated that P28-loaded 7.5% HAp/FAp had better performance in the swelling study, porosity, and osteogenic induction, indicating it is a promising bone scaffold candidate; however, future studies should focus on optimizing the balance between porosity and mechanical strength. Nevertheless, this study has limitations, including the lack of release kinetics evaluation and the possibility of uncontrolled burst release of VEGF/P28, which may have influenced its sustained bioavailability during culture. In addition, the absence of microscopic images to directly visualize scaffold morphology, swelling, degradation, and cell–scaffold interactions limited the ability to fully confirm the quantitative observations. Future work will focus on characterizing release behavior, developing controlled or sequential delivery strategies, and incorporating SEM or fluorescence microscopy to provide complementary morphological evidence and better promoted osteogenic processes.

## Figures and Tables

**Table 1:** Scaffold formulations with varying *w*/*v*% of CS pastes and HAp/FAp ratios.

Scaffold ID	HAp (%wt)	FAp (%wt)	Volume of 1% *v*/*v* AA/(mL)	PEGDMA (µL)
5% HAp/FAp	0.75	0.75	30	100
7.5% HAp	1.5	0	20	100
7.5% HAp/FAp	0.75	0.75	20	100
12% HAp	1.5	0	12.5	100

**Table 2:** Ion concentration of simulated body fluid (SBF) and human blood plasma [87].

	Ion Concentration (mM)							
	Na^+^	K^+^	Mg^2+^	Ca^2+^	Cl^−^	HCO_3_^−^	HPO_4_^2−^	SO_4_^2−^
SBF	142.0	5.0	2.5	1.5	148.8	4.2	1.0	0.5
Human plasma	142.0	5.0	2.5	1.5	103.0	27.0	1.0	0.5

**Table 3:** Primer sequences used for real-time RT-qPCR.

*Gene*	*Primer Sequences Forward/Reverse*
***Bglap***	5′-GACACCATGAGGACCATCTTTC–3′/5′-CATGAAGGCTTTGTCAGACTCA–3′
***Col1a1***	5′–CCAATGGTGCTCCTGGTATT–3′/5′–GGTTCACCACTGTTACCCTT–3′
***Runx2***	5′–CTCTGATCGCCTCAGTGATTT–3′/5′–CTGCCTGGGATCTGTAATCTG–3′
***Tbp***	5′–AGTGCCCAGCATCACTATTT–3′/5′–GGTCCATGATTCTCCCTTTCTT–3′

## Data Availability

Data are contained within the article.

## Abbreviations

The following abbreviations are used in this manuscript:

AA	Acetic acid
ALP	Alkaline phosphatase activity
BMP-2	Bone morphogenetic protein 2
BTE	Bone tissue engineering
CCK–8	Cell counting kit–8
CS	Chitosan
ECM	Extracellular matrix
EWC	Equilibrium water content
FAp	Fluorapatite
FBS	Fetal Bovine Serum
GF	Gel fraction
HAp	Hydroxyapatite
*Runx2*	Runt-related transcription factor 2
RT-qPCR	Reverse transcription-quantitative polymerase chain reaction
SBF	Simulated body solution
VEGF	Vascular endothelial growth factor
